# Cytotoxicity and Antioxidant Potential of Novel 2-(2-((1*H*-indol-5yl)methylene)-hydrazinyl)-thiazole Derivatives

**DOI:** 10.3390/molecules22020260

**Published:** 2017-02-09

**Authors:** Adriana Grozav, Ioan-Dan Porumb, Luiza Ioana Găină, Lorena Filip, Daniela Hanganu

**Affiliations:** 1 Department of Organic Chemistry, Faculty of Pharmacy, “Iuliu Haţieganu” University of Medicine and Pharmacy, 8 Victor Babes, Cluj-Napoca RO-400012, Romania; adriana.ignat@umfcluj.ro; 2 Research Center on Fundamental and Applied Heterochemistry, Faculty of Chemistry and Chemical Engineering, “Babeş-Bolyai”, University, M. Kogalniceanu 1, Cluj-Napoca RO-400028, Romania; pdan@chem.ubbcluj.ro; 3 Department of Bromatology, Hygiene, Nutrition, Faculty of Pharmacy, “Iuliu Haţieganu” University of Medicine and Pharmacy, 8 Victor Babes, Cluj-Napoca RO-400012, Romania; 4 Department of Pharmacognosy Faculty of Pharmacy, “Iuliu Haţieganu” University of Medicine and Pharmacy, 8 Victor Babes, Cluj-Napoca RO-400012, Romania; dhanganu@umfcluj.ro

**Keywords:** cytotoxic activity, antioxidant activity, DFT calculation, indol, thiazole

## Abstract

Newly synthesized 2-(2-((1*H*-indol-5yl)methylene)-hydrazinyl)-thiazole derivatives were evaluated for their in vitro cytotoxicity on two carcinoma cell lines A2780 and HeLa. Significant cytotoxic activity for 2-(2-((1*H*-indol-5-yl)methylene)hydrazinyl)-4-methylthiazole (**1**) and 2-(2-((1*H*-indol-5-yl)methylene)hydrazinyl)-4-phenylthiazole (**3**), on both A2780 [IC_50_: 11.6 μM (**1**), and 12.4 μM (**3**)] and HeLa [IC_50_: 22.4 μM (**1**) and 19.4μM (**3**)] cell lines is reported. Their antioxidant potential was evaluated by spectrophotometric method, using DPPH radical or Fe (TPTZ)^3+^ complex, and EPR spectroscopy, therefore the compounds **1** and **3** showed remarkable antioxidant activity simultaneously with a cytotoxic effect on A2780 and HeLa cell lines. Furthermore, based on theoretical quantum chemical calculation, the present study analyzed the chemoselectivity of the hydrogen extraction from the indolyl-hydrazinil-thiazoles in reaction with free radicals.

## 1. Introduction

The heterocyclic derivatives with thiazole or indole units can be found in a variety of biologically active compounds that makes them some of the most extensively studied heterocycles. An example of a natural compound in which both indole and thiazole rings are linked is 3-thiazol-2′-yl-indole known as Camalexin, a phytoalexin produced in different plant leaves [1,2] or synthetic derivatives based on thiazolyl-1*H*-indols with antiproliferative activity in peritoneal mesothelioma or MiaPaCa-2 cell line [3,4], HIV-1 RT inhibitors based on thiazolyl-hydrazinyl-1*H*-indol-2-one derivatives [5] and isomer analogs of our compounds acting as inhibitors for *Mycobacterium tuberculosis* [6].

The recent investigations are focused to develop new molecules based on indole scaffolds because of their biological potential, essential amino acid like tryptophan, psychoactive substances [7] like synthetic alkaloids, Sumatriptan for migraine treatment, Vincristine and other indole derivatives as antiproliferative agents [8], Delavirdine anti-HIV drug, Doleasetron antiemetic drug [9], and antioxidant compounds [10] but the examples of biologically active compounds bearing indole ring are in continuous expansion.

The thiazole can be identified in the structure of different drugs, epothilone a microbial product and their synthetic analogs having anti-cancer properties, thiamine is vitamin B1, sulfathiazolan is antimicrobial agent, abafunginas is fungicide, tiazofurin an antineoplastic, ritonavir is anti-HIV, and fanetizole, meloxicam, and fentiazacare are anti-inflammatory agents [11,12].

Thiazoles bearing hydrazine moieties have gained a special attention because of their biological and pharmacological properties as inhibitors on human monoamine oxidase (hMAO) [13], antimicrobial agents [14], histone acetyltransferase inhibitors [15], anti-inflammatory agents [16], as well as antiproliferative activity highlighted by our previous studies [17,18,19].

Considering the biological potential of heterocyclic derivatives bearing thiazole and indole scaffolds, the objectives of this study were to identify new possible antiproliferative agents and the study of their antioxidant potential. For a better understanding of the antioxidant properties on molecular level, the structure of 2-(2-((1*H*-indol-5-yl)methylene)-hydrazinyl)-thiazole derivatives and the chemoselectivity of the radical generation reactions have been studied using molecular modeling tools as Density Functional Theory (DFT) using hybrid B3LYP, 6-31G*, and Hartree-Fock 3-21G models [20,21,22] offered by Spartan 06 software.

## 2. Results and Discussion

### 2.1. Synthesis of the New 2-(2-((1H-Indol-5-yl)methylene)hydrazinyl)thiazole Derivatives

**Scheme 1:**
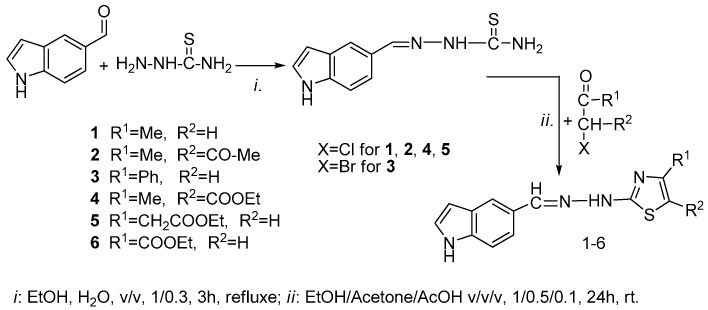
Synthesis of 2-(2-((1*H*-indol-5-yl)methylene)hydrazinyl)thiazole derivatives.

The synthesis of newly 2-(2-((1*H*-indol-5-yl)methylene)-hydrazinyl)-thiazole derivatives were performed by the Hantzsch protocol in two steps, the condensation of 1*H*-indole-5-carbaldehyde with hydrazinecarbothioamide yielding (*E*)-2-[(1*H*-indol-3-yl)methylene] thiosemicarbazone [23] followed by its cyclocondensation with α-halocarbonyl derivatives, Scheme 1.

The structure of indolyl-hydrazinyl-thiazole derivatives **1**–**6** were confirmed by NMR, spectroscopy (^1^H-NMR and ^13^C-NMR, COSY, HMQC and HMBC) and mass spectrometry. The characteristic singlet for imine proton (HC=N) can be identified around 8.06–8.41 ppm, while the NH proton from indole ring appears around 10.6 ppm in acetone but more deshielded, around 11.5 ppm, in a more polar solvent like dimethyl sulfoxide*.*

### 2.2. Cytotoxic Activity

**Figure 1:**
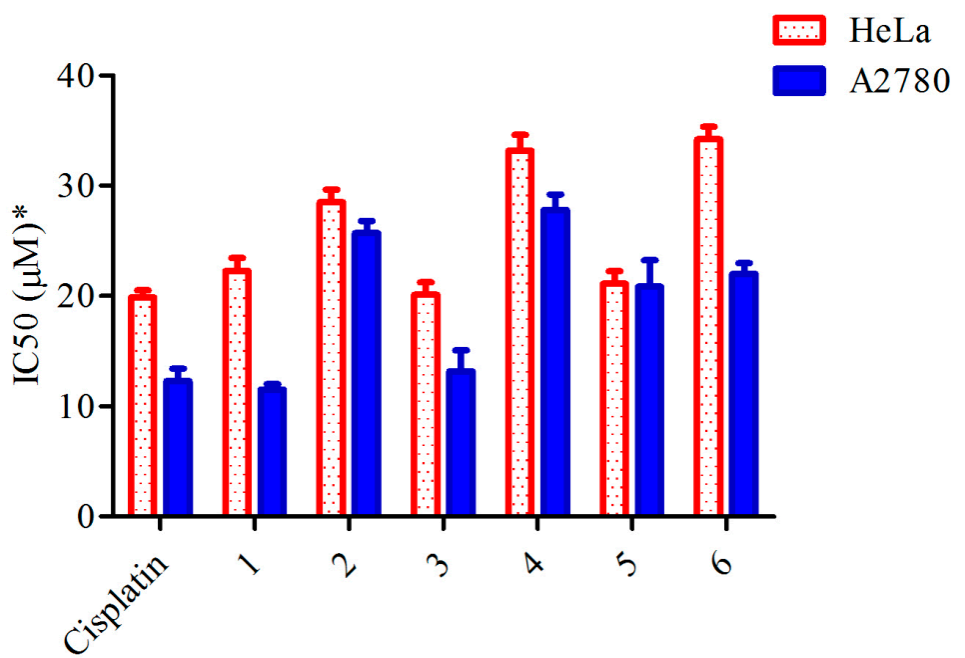
Cytotoxic activity of in vitro of Compounds **1**–**6** against HeLa and A2780 tumor cells. * Data are reported as IC_50_ values determined by MTT assay after 24 h of continuous exposure to each compound. The values were calculated using sigmoidal dose-response relationship. The data represents mean values ± SEM (standard error of mean) of at least three independent experiments.

The new indolyl-hydrazinyl-thiazoles **1**–**6** were tested in vitro, by colorimetric method MTT, for their cytotoxic activity on two tumor cell lines: HeLa (human cervical cancer cells) and A2780 (human ovarian cancer cells). The inhibitory activity of the compounds was measured and quantified using the mathematic parameter IC_50_, (Figure 1). Cisplatin, a metal-based drug currently used in cancer therapy, was used as control (Figure 1).

The in vitro results displayed a better cytotoxic activity of indolyl-hydrazinyl-thiazoles **1**–**6** on tumor line A2780, the IC_50_ values being lower compared with those obtained on HeLa tumor line.

Compounds **1** and **3** displayed cytotoxic activity comparable to cisplatin on both tumor cell lines while the cytotoxic activity displayed by compounds **4** and **6** appeared inferior to the standard on both tumor cell lines. In comparison with cisplatin standard, compound **5** has a similar activity on HeLa cell lines, but it proved to be less sensitive on A2780 cell lines.

### 2.3. Antioxidant Activity

The spectrophotometric method based on free radical scavenging activity is widely used to conveniently assess antioxidant potential in vitro. The scavenging potential of the indolyl-thiazole derivatives, in terms of hydrogen donating ability, was evaluated using DPPH (1,1-Diphenyl-2-picrylhydrazyl) radicals. The scavenging potential of the indolyl-hydrazinyl-thiazole derivatives in the presence of DPPH decreases as follows **1** > **3** > **2** > **5** > **4** > **6**, Table 1. The antioxidant potential of the compounds **1** (IC_50_ = 5.377 µg/mL) and **3** (IC_50_ = 9.131 µg/mL) is better than trolox-standard (IC_50_ = 9.74 µg/mL) but lower than ascorbic acid (IC_50_ = 2.46 µg/mL) activity.

The antioxidant potential of thiazols **1**–**6** towards free DPPH radicals was also investigated by Electron Paramagnetic Resonance (EPR) spectroscopy. The EPR spectra have shown that the integral intensity of DPPH radicals in the mixture with these antioxidant compounds decreases compared to free DPPH solution, (I = 252) and represents the oxido-reduction rate. The order of reactivity based on the calculated rates is identical to the one found by DPPH spectrophotometric method, as seen in Table 1.

The reducing capacity of indolyl-hydrazinyl-thiazoles **1**–**6** has been measured as ferric reducing antioxidant power (FRAP). The reduction of Fe^3+^ ion from tripyridyltriazine Fe (TPTZ)^3+^ complex to Fe^2+^ ion in the blue colored Fe (TPTZ)^2+^ complex depends on the electron donating capacity of the tested thiazoles **1**–**6** and was quantified by spectrophotometric method. The reducing ability of the compounds decreases as follows **1** > **3** > **5** > **4** > **6** > **2** (see Table 1). According to the results obtained by spectrophotometric methods using DPPH radical, Fe (TPTZ)^3+^ complex and EPR spectroscopy, Compound **1** is the most active from the newly synthesized thiazole derivatives **1**–**6**.

These new indolyl-hydrazinyl-thiazoles displayed both antioxidant activity and cytotoxicity against human carcinoma cell lines A2780 and HeLa. Thus, they could be further evaluated as prophylactic agents usable in preventing the free radical induced chain reactions with repercussions in tumoral cell growth.

### 2.4. Computational Study

The quantum chemical calculations for novel synthesized 2-(2-((1*H*-indol-5-yl)methylene)-hydrazinyl)-thiazole derivatives **1**–**6** were performed using Spartan 06 software (Wavefunction Inc., Irvine, CA, USA). The quantum molecular parameters such as energies corresponding to optimized geometries (E) and frontier molecular orbitals (E_HOMO_, E_LUMO_), were computed using Density Functional Theory (DFT), hybrid B3LYP, 6-31G*. Table 2 summarizes representative computational results.

The antioxidant activities of indolyl-hydrazinyl-thiazoles **1**–**6** were experimentally investigated by three method involving two different reaction pathways, the first mechanism was based on hydrogen donation during the reaction with DPPH radicals and the second method was based on the electron donation to the Fe^+3^ ion in a reducing reaction known as FRAP method. For a better understanding of the radical scavenging abilities on a molecular level, the chemoselectivity of radical generation was investigated by theoretical quantum chemical calculations.

During the reaction of DPPH radical with thiazoles **1**–**6**, these are converted in radicals by losing one hydrogen atom. As the indolyl-hydrazinyl-thiazoles **1**–**6** contain two different NH units there are two possible ways to generate radicals; although the extraction of the hydrogen from the hydrazinyl bridge seems to have a higher probability, the possibility to extract hydrogen atom from the indole moiety can be taken into consideration. The analysis of this issue was performed using molecular mechanics and quantum chemical calculations.

The HOMO orbital from indolyl-hydrazinyl-thiazoles **1**–**6**. Table 2, is mainly located on the hydrazinyl bridge and less in the indole group, favoring the electron or hydrogen atom extraction from the hydrazinyl moiety, Table 3.

**Scheme 2:**
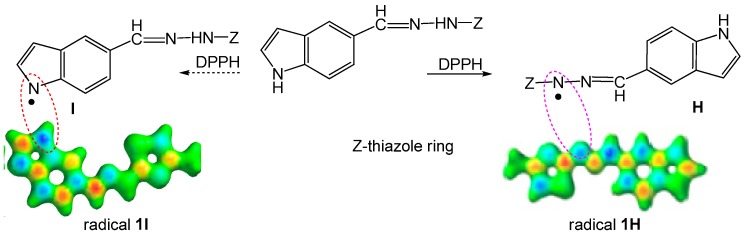
The structure of two possible radicals of 2-(2-((1*H*-indol-5-yl)methylene)-hydrazinyl)-thiazole **1** located on hydrazinyl (**H**), respective on indolyl (**I**) moiety and spin density map for radicals **H** and **I** type.

This supposition was confirmed by the values of the energies corresponding to optimized geometries for both possible radical **H** ad **I** types, as seen in Table 3 and Scheme 2.

The radicals belonging to the thiazoles **1**–**6** were generate from optimized geometries, previously calculated by DFT B3LYP, 6-31G* and computed using MMFT molecular mechanics to establish equilibrium conformation. The semi-empirical AM1 model was used to establish equilibrium geometry and Hartree-Fock 3-21G model for a basis of graphical calculation.

Comparing the calculated energies (E) for both type of radicals **I** and **H** we found that the **H** type radicals have a lower energy than **I** type radicals, Table 3. The energy gap (E_I_–E_H_) between radicals **H** and **I** was around 15–29 kcal/mol, enough to generate predominantly **H** radicals from all thiazoles **1**–**6**. The **H** type radicals can be stabilized by internal conjugation with indole and thiazole ring and the favored pathway for spin delocalization is on indolyl-methylene moiety, in respect with SOMO orbital, Table 3.

In the spin density map the blue color on the surface indicates maximum electron density that decreases from blue to red color. As expected, in each case the indolyl-methylene unit contains a high electron density located on the methylene atom C^5^.

## 3. Experimental Section 

### 3.1. Materials and Methods

For the chemical synthesis the reagents and solvents were bought from chemical suppliers and used without further purification. 2,4,6-Tripyridyl-s-triazine (TPTZ), iron (III) chloride (FeCl_3_·6H_2_O), 2,2-diphenyl-1-picryl hydrazyl (DPPH) and standards as 6-hydroxy-2,5,7,8-thetramethylchroman-2-carboxilyc acid (Trolox) were purchased from Sigma-Aldrich Chemie Gmbh (Munich, Germany). 

The tumor cells were grown in the presence of RPMI 1640 (Sigma-Aldrich), fetal calf serum (FCS, Sigma-Aldrich, 5%), glutamine (Sigma-Aldrich, 0.1%), and antibiotics penicillin–streptomycin ActavisSindanPharma (Bucharest, Romania).

The ^1^H-NMR and ^13^C-NMR spectra were recorded in acetone-*d*_6_, or DMSO-*d*_6_ (locked to Me_4_Si) using a 400MHz Bruker Avance NMR spectrometer produced by Bruker BioSpin GmbH (Rheinstetten, Germany). The mass spectra were recorded on Thermo Scientific LTQ Orbitrap XL mass spectrometer produced by ThermoFisher Scientific (Bremen, Germany). Melting points were measured with an Electrothermal IA 9200 apparatus produced by Cole-Parmer (Staffordshire, UK), and are uncorrected values. Colorimetric measurements were recorded with Biotek Synergy 2 Multi-Mode Microplate Reader with SQ Xenon Flash light source Produced by BioTek (Winooski, VT, USA). The absorbance has been measured using a Jasco V-530 UV-Vis spectrophotometer produced by Jasco International Co. Ltd. (Tokyo, Japan). Electron paramagnetic resonance measurements were performed on a BrukerElexsys E500 spectrometer (Bruker, Billerica, MA, USA) operating in X band (~9.4 GHz) with 100 kHz modulation frequency; the parameters of scanning are: center field, 3360 G; sweep width, 60 G; power, 2 mW; receiver gain, 1 × 103; modulation amplitude, 2 G; time of conversion, 15 ms; time constant, 30.72 ms; sweep time 60 s.

### 3.2. Chemistry

#### 3.2.1. General Procedure for (*E*)-2-[(1*H*-Indol-3-yl) methylene]thiosemicarbazone

To a solution of 1*H*-indole-5-carbaldehyde (14.5 g, 0.1 mol) in ethanol (100 mL) was added a solution of hydrazine carbothioamide (9.1 g, 0.1mol) in water (30 mL). The solution was stirred under reflux for 5 h. The reaction progress was monitored by TLC (toluene/AcOEt 2/1 *v*/*v*; silica plates), after the reaction completed the solid phase was filtered and recrystallized from ethanol obtaining a light brown powder of thiosemicarbazone (15 g, 78%, m.p. 217 °C, m.p. 217–218 °C lit. [23]).

#### 3.2.2. General Procedure for Compounds **1**–**6**

A mixture of (*E*)-2-[(1*H*-indol-3-yl)methylene]thiosemicarbazone (0.01 mol) and α-halogenocarbonyl derivative (0.01 mol) in ethanol/acetone/acetic acid (20 mL, 1/0.5/0.1 *v*/*v*/*v*) was stirred at room temperature for 24 h. The reaction progress was monitored by TLC (toluene/AcOEt 2/1 *v*/*v*; silica plates), after the reaction completed, the mixture was neutralized at pH 7 with NaHCO_3_ aqueous solution (10%). The precipitate was filtered and recrystallized from ethanol, derivatives **4** and **6** were purified by column chromatography (silicagel 60, eluent toluene/acetone 4/1 *v*/*v*).

*2-(2-((1H-Indol-5-yl)methylene)hydrazinyl)-4-methylthiazole* (**1**)


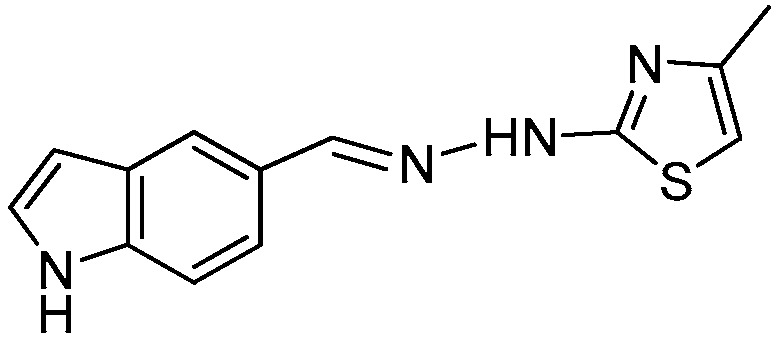


Light gray powder, yield 2.0 g, 79%, m.p. 182 °C, crystallized from ethanol; ^1^H-NMR (DMSO-*d*_6_, 400 MHz) δ (ppm): 2.16 (s, 3H), 6.33 (s, 1H), 6.48–6.49 (m, 1H), 7.37–7.38 (m, 1H), 7.42 (d, 1H, *J* = 8.5Hz), 7.49 (dd, 1H, *J* = 1.3 Hz, *J* = 8.5 Hz), 7.37 (s, 1H), 8.06 (s, 1H), 11.27 (s, 1H), 11.56 (br, 1H); ^13^C-NMR (DMSO-*d*_6_, 100 MHz) δ (ppm): 17.6, 102.2, 104.0, 112.4, 119.2, 120.3, 126.2q, 126.7, 128.1q, 136.9q, 143.8, 155.3q, 168.6q. HRMS (APCI) calcd. for C_13_H_13_N_4_S [M + H]: 257.0861 found 257.0812.

*1-(2-(2-((1H-Indol-5-yl)methylene)hydrazinyl)-4-methylthiazol-5-yl)ethanone* (**2**)


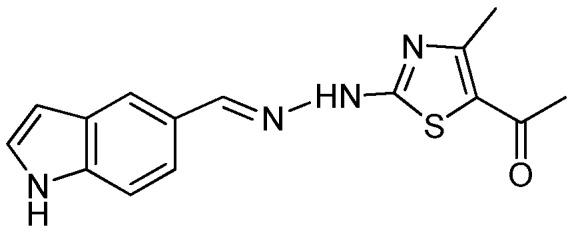


Sand-colored powder, yield 2.4 g, 82%, m.p. 256 °C, crystallized from ethanol; ^1^H-NMR (acetone-*d*_6_, 400 MHz) δ (ppm): 2.44 (s, 3H), 2.53 (s, 3H), 3.66 (br, 1H), 6.57–6.58 (m, 1H), 7.39–7.41 (m, 1H), 7.25 (d, 1H, *J* = 8.6 Hz), 7.66 (d, 1H, *J* = 8.6 Hz), 7.88 (s, 1H), 8.27 (s, 1H), 10.60 (br, 1H); ^13^C-NMR (acetone-*d*_6_, 100 MHz) δ (ppm): 19.6, 29.6, 101.1, 104.0, 111.7, 119.6, 120.9, 126.0, 128.3q, 130.5q, 132.4q, 146.9, 156.0q, 171q, 189.4q. HRMS (APCI) calcd. for C_15_H_15_N_4_OS [M + H]: 299.0967, found 299.0911.

*2-(2-((1H-Indol-5-yl)methylene)hydrazinyl)-4-phenylthiazole* (**3**)


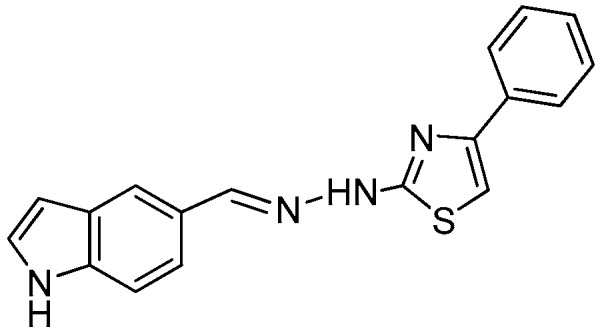


Light brown powder yield 2.3 g, 75%, m.p. 246 °C, crystallized from ethanol; ^1^H-NMR (DMSO-*d*_6_, 400 MHz) δ (ppm): 6.52 (s, 1H), 7.34 (s, 2H), 7.41–7.45 (m, 3H), 7.51 (d, 1H, *J* = 8.5 Hz), 7.60 (d, 1H, *J* = 8.5 Hz), 7.74 (d, 2H, *J* = 7.4), 7.86 (s, 1H), 8.41 (s, 1H), 11.47 (s, 1H), 12.01 (br, 1H); ^13^C-NMR (DMSO-*d*_6_, 100 MHz) δ (ppm): 102.6, 104.5, 112.6, 119.7, 121.6, 125.1, 126.4q, 126.5 (2C), 127.0q, 128.1, 129.2 (2C), 130.2q, 137.5, 148.5, 154.6q, 168.8q. HRMS (APCI) calcd. for C_18_H_15_N_4_S [M + H]: 319.1017, found 319.0959.

*Ethyl 2-(2-((1H-Indol-5-yl)methylene)hydrazinyl)-4-methylthiazole-5-carboxylate* (**4**)


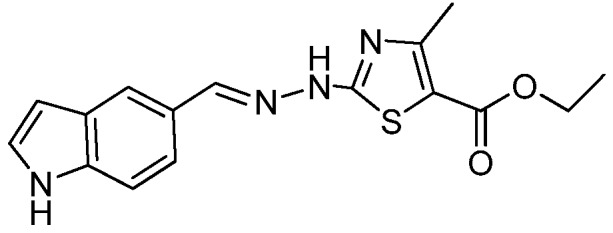


Light gray powder, purified by column chromatography, yield 1.9 g, 60%, m.p. 265 °C, from toluene/acetone; ^1^H-NMR (DMSO-*d*_6_, 400 MHz) δ (ppm):1.27 (t, 3H, *J* = 7.1Hz), 2.47 (s, 3H), 4.22 (q, 2H, 7.1), 6.50–6.51 (m, 1H), 7.39–7.40 (m, 1H), 7.45 (d, 1H, *J* = 8.5 Hz), 7.54 (d, 1H, *J* = 8.5 Hz), 7.80 (s, 1H), 8.16 (s, 1H), 11.33 (s, 1H), 12.28 (br, 1H). ^13^C-RMN: (DMSO-*d*_6_, 100 MHz) δ (ppm): 14.8, 17.4, 60.5, 102.4, 108.9Cq, 112.5, 119.5, 121.1, 125.6Cq, 126.7, 128.1Cq, C137.3Cq, 146.9, 158.1Cq, 162.9Cq, 169.7Cq. HRMS (APCI) calcd. for C_16_H_17_N_4_O_2_S [M + H]: 329.1072, found 329.1013.

*Ethyl 2-(2-(2-((1H-Indol-5-yl)methylene)hydrazinyl)thiazol-4-yl)acetate* (**5**)


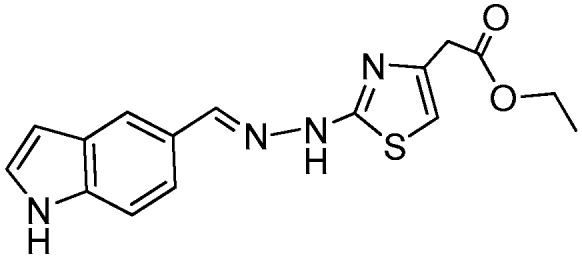


Light brown powder yield 1.9 g, 60%, m.p. 191 °C, crystallized from ethanol; ^1^H-NMR (acetone-*d*_6_, 400 MHz) δ (ppm): 1.33 (t, 3H, *J* = 7.1 Hz), 3.05 (br, 1H), 3.58 (s, 2H), 4.22 (q, 2H, *J* = 7.1 Hz), 6.54–6.55 (m, 1H), 6.59 (s, 1H), 7.37–7.38 (m, 1H), 7.49 (d, 1H, *J* = 8.5 Hz), 7.63 (dd, 1H, *J* = 8.5Hz, *J* = 1.4 Hz), 8.19 (s, 1H), 10.61 (br, 1H); ^13^C-NMR (acetone-*d*_6_, 100 MHz) δ (ppm): 13.5, 37.0, 60.1, 102.1, 104.9, 111.8, 119.3, 120.2, 125.8, 126.4q, 128.2q, 137.0q, 143.6, 145.6q, 170q, 189.4q. HRMS (APCI) calcd. for C_16_H_17_N_4_O_2_S [M + H]: 329.1072, found 329.1013.

*(E)-Ethyl 2-(2-((1H-Indol-5-yl)methylene)hydrazinyl)thiazole-4-carboxylate* (**6**)


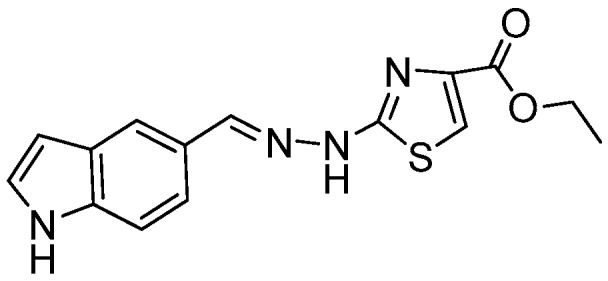


Light brown powder, purified by column chromatography, yield 1.4 g, 45%, m.p. 210 °C, from toluene/acetone; ^1^H-NMR (DMSO-*d*_6_, 400 MHz) δ (ppm):1.29 (t, 3H, *J* = 6.8 Hz), 4.24 (q, 2H, *J* = 6.8 Hz), 6.49–6.50 (m, 1H), 7.38–7.39 (m, 1H), 7.45 (d, 1H, *J* = 8.8 Hz), 7.52 (d, 1H, *J* = 8.8 Hz), 7.72 (s, 1H), 7.77 (s, 1H), 8.08 (s, 1H), 11.30 (s, 1H), 12.06 (br, 1H). ^13^C-NMR: (DMSO-*d*_6_, 100 MHz) δ (ppm): 14.8, 60.5, 102.3, 108.5Cq, 112.5, 119.2, 120.6, 125.8Cq, 126.8, 128.1Cq, C137.1Cq, 144.6, 161.5, 164.1Cq, 168.7Cq. HRMS (APCI) calcd. for C_15_H_15_N_4_O_2_S [M + H]: 315.0916, found 315.0965.

### 3.3. Biology

#### 3.3.1. Cell Cultures

The human cervical cancer cells (HeLa) and the human ovarian cancer cells (A2780) were obtained from the European Centre of Cell Cultures (ECACC—Sigma Aldrich, Munich, Germany). These cell lines were cultivated under sterile conditions by using RPMI 1640 growth media supplemented with fetal calf serum (FCS), glutamine, and antibiotics (penicillin—streptomycin, 0.1%) at 37 °C and CO_2_ (5%)

##### Cell Proliferation Inhibition

The MTT assay was used in order to determine the cytotoxicity. The cells were seeded in 96-well plates with 100 μL of cell solution (ca. 10.000 cells/well) and incubated for 24 h. The compounds **1**–**6** were initially dissolved in DMSO, followed by a series of successive dilutions using RPMI 1460 media, so that the final concentration of DMSO was under 0.1%.

The cells were treated with indolyl-hydrazinyl-thiazoles derivatives (concentrations between 0.1 µM–100 µM) for 24 h. After this treatment, we removed the culture media and we added MTT (3-(4,5-dimethyl-2-thiazolyl)-2,5-diphenyl-2*H*-tetrazolium bromide) Hanks media solution in each well. Then, the plates were incubated for 2 h. The formazan crystals that were formed by the mitochondrial dehydrogenase activity of vital cells were dissolved in DMSO.

The optical density, an indicator of cellular viability, was quantified by colorimetric measurements and it is directly proportional to the amount of formazan crystals formed in the cells. A multimode microplate reader was used to measure the plates and the absorbance was detected at 570 nm.

Note: As a positive control in the experiment, we used cisplatin drugs, in the same concentrations of the studied compounds. All the experiments were performed in triplicate.

##### Statistical Analysis

Values are given as the mean ± SEM. The data, performed in triplicate, are represented as averages of independent experiments. Graph Pad Prism 5 biostatistics software was used in order to process the experimental data.

#### 3.3.2. Antioxidant Activity

##### DPPH Based Free Radical Scavenging Activity

The antioxidant potential of the indolyl-hydrazinyl-thiazoles **1**–**6** was measured in reaction with DPPH radical and quantified using a spectrophotometric method. The color change of DPPH radical solution from purple to yellow in the presence of indolyl-hydrazinyl-thiazoles **1**–**6**, generated a decrease of absorbance intensity at 517 nm.

A DPPH methanolic solution (200 μL, 0.1 g/L) was mixed with a methanolic solution of one of the tested compounds **1**–**6** as follows **1** (1.6 μg/mL), **2** (1.8 μg/mL), **3** (1.9 μg/mL), **4** (2 μg/mL), **5** (2 μg/mL), and **6** (2.4 μg/mL) and then each sample volume was adjusted up to 10 mL with methanol. The absorbance was measured at 517 nm after the samples were incubated for 30 min at 40 °C [24].

The radical scavenging ability for the tested indolyl-hydrazinyl-thiazoles **1**–**6** was calculated as a percentage of absorbance intensity: radical scavenging ability (%) = [(A_c_ − A_s_)/A_c_] × 100, where A_c_ is the absorbance of DPPH radical solution and A_s_ is the absorbance of the solution containing DPPH radical mixed with tested compound. The scavenging activity of Trolox and ascorbic acid were measured and compared with the one of the tested compounds. Antiradical activity has been expressed as IC_50_, the concentration (μg/mL) of the tested compounds required for a 50% DPPH**^●^** inhibition.

##### Reducing Power by Ferric Reducing Antioxidant Power (FRAP) Test

The solutions of the tested indolyl-hydrazinyl-thiazoles **1**–**6** have been prepared in the range of 1.35–1.64 mg/mL concentration. The samples were prepared by mixing of “FRAP” reagent see lit [25]. (6 mL) with H_2_O (0.4 mL) and indolyl-hydrazinyl-thiazole (0.4 mL). After 10 min the absorbance has been measured at 593 nm and the antioxidant capacity has been expressed by µM Trolox equivalent/g.

##### Electron Paramagnetic Resonance (EPR) Spectroscopy Method

In EPR quartz capillary is inserted a mixture of DPPH methanolic solution (10 μL, 4.5 mmol) and indolyl-hydrazinyl-thiazole methanolic solution (10 μL, 5mmol) and EPR spectra were recorded at room temperature. 

The relative concentration of paramagnetic species were determined by a double integration of experimental spectra using XEPR Bruker software [26,27].

## 4. Conclusions

A new series of 2-(2-((1*H*-Indol-5-yl) methylene)-hydrazinyl)-thiazole derivatives, **1**–**6**, has been synthesized in moderate to good yields by a Hantzsch protocol and their structures were confirmed by NMR spectroscopy and mass spectrometry. The in vitro cytotoxicity was evaluated for all indolyl-hydrazinyl-thiazoles **1**–**6** on human cervical cancer cells (HeLa) and human ovarian cancer cells (A2780); Compounds **1** and **3** had a good inhibition on both A2780 and HeLa while derivative **5** has been active only on HeLa cell lines.

The antioxidant potential of newly synthesized thiazoles **1**–**6** was studied by three different methods, in two of these the radical scavenging properties were tested and one was based on their capacity to act as reducing agent. According to these results, derivatives **1** and **3** were found to be promising antioxidant agents.

The chemoselectivity of radicals formation in reaction of 2-(2-((1*H*-indol-5-yl)methylene)-hydrazinyl)-thiazoles **1**–**6** with free DPPH radicals was investigated using theoretical quantum chemical calculation; based on this study the hydrogen atom is extracted from hydrazinyl bridge rather than from indole moiety.

## Figures and Tables

**Table:** 

Compounds	IC_50_ (μM)	
	HeLa	A2780
Cisplatin	20.10 ± 0.22	12.30 ± 0.22
**1**	22.42 ± 0.24	11.65 ± 0.21
**2**	28.55 ± 0.25	26.49 ± 0.21
**3**	19.42 ± 0.22	12.99 ± 0.50
**4**	33.98 ± 0.31	28.08 ± 0.23
**5**	19.84 ± 0.33	19.66 ± 0.50
**6**	34.72 ± 0.25	20.15 ± 0.26

**Table 1:** The antioxidant activity of indolyl-hydrazinyl-thiazoles **1**–**6** determined by different methods.

Compounds	Methods		
	DPPH IC 50 (µg/mL)	FRAP µM ET/g	EPR Integral Intensity Value
**1**	5.377 ± 0.52	9835.5518 ± 324	17 ± 0.14
**2**	11.850 ± 0.72	872.5288 ± 27	72 ± 0.23
**3**	9.131 ± 0.61	8176.5278 ± 298	24 ± 0.17
**4**	13.357 ± 0.68	6129.6298 ± 280	114 ± 0.3
**5**	12.974 ± 0.58	7431.0798 ± 263	82 ± 0.52
**6**	15.658 ± 0.63	3572.2890 ± 124	142 ± 0.34
Trolox	9.74 ± 0.24	-	-
Ac.ascorbic	2.46 ± 0.91	-	-

**Table 2:** Quantum molecular parameter and frontier orbital density distribution for indolyl-hydrazinylthiazoles **1**–**6**.

Compounds	E_HOMO_ (eV)	E_LUMO_ (eV)	E_HOMO-LUMO_ (eV)	HOMO Surface
**1**	−4.96	−0.98	3.98	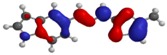
**2**	−5.29	−1.52	3.77	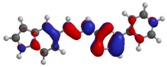
**3**	−5.00	−1.10	3.90	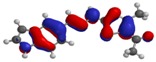
**4**	−5.22	−1.38	3.84	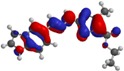
**5**	−4.96	−0.96	4.00	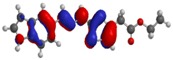
**6**	−5.23	−1.20	4.03	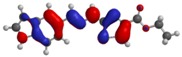

**Table 3:** The quantum molecular parameter and spin density distribution for **H** type radicals generated from indolyl-hydrazinylthiazoles **1**–**6**.

Radical		E (kcal/mol)	E_HOMO_ (eV) H_radical_	E_LUMO_ (eV) H_radical_	Spin Density Map for H_radical_	Spin Density on C^5^ for H_radical_ (eV) *	SOMO Orbital for H_radical_
**1**	H	−695,328.05	−7.94	3.09	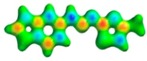	0.035	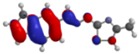
	I	−695,310.80					
**2**	H	−790,033.74	−8.08	2.54	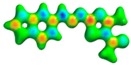	0.036	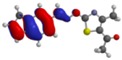
	I	−790,016.36					
**3**	H	−814,214.89	−7.98	2.91	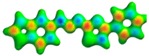	0.037	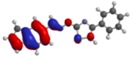
	I	−814,185.61					
**4**	H	−861,114.17	−8.06	2.59	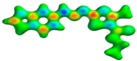	0.036	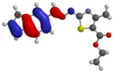
	I	−861,098.95					
**5**	H	−861,108.82	−7.97	2.98	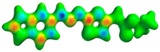	0.038	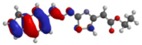
	I	−861,085.82					
**6**	H	−836,749.36	−8.07	2.20	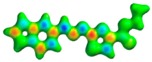	0.037	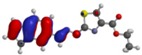
	I	836,728.71					

* The spin density for radical **H** on C^5^atom (indole-C^5^H=N-NH-thiazole) represent the value at the cursor point and the blue color on the surface indicates maximum electron density on that atoms.

## References

[1] Browne L.M., Conn K.L., Ayert W.A., Tewari J.P. (1991). The camalexins: New phytoalexins produced in the leaves of *camelinasativa* (cruciferae). Tetrahedron.

[2] Glawischnig E. (2007). Camalexin. Phytochemistry.

[3] Carbone A., Pennati M., Parrino B., Lopergolo A., Barraja P., Montalbno A., Spanò V., Sbarra S., Doldi V., de Cesare M. (2013). Novel 1*H*-pyrrolo[2,3-*b*]pyridine derivatives nortopsentin analogues: Synthesis and antitumor activity in peritoneal mesothelioma experimental models. J. Med. Chem..

[4] Diana P., Carbone A., Barraja P., Montalbano A., Parrino B., Lopergolo A., Pennati M., Zaffaroni N., Cirrincione G. (2011). Synthesis and antitumor activity of 3-(2-phenyl-1,3-thiazol-4-yl)-1*H*-indoles and 3-(2-phenyl-1,3-thiazol-4-yl)-1*H*-7-azaindoles. ChemMedChem.

[5] Meleddu R., Distinto S., Corona A., Bianco G., Cannas V., Esposito F., Artese A., Alcaro S., Matyus P., Bogdan D. (2015). (3*Z*)-3-(2-[4-(aryl)-1,3-thiazol-2-yl]hydrazin-1-ylidene)-2,3-dihydro-1*H*-indol-2-one derivatives as dual inhibitors of HIV-1 reverse transcriptase. Eur. J. Med. Chem..

[6] Makam P., Kankanala R., Prakash A., Kannan T. (2013). 2-(2-Hydrazinyl)thiazole derivatives: Design, synthesis and in vitro antimycobacterial studies. Eur. J. Med. Chem..

[7] Kondrasenko A.A., Goncharov E.V., Dugaev K.P., Rubaylo A.I. (2015). CBL-2201. Report on a new designer drug: Napht-1-yl 1-(5-fluoropentyl)-1*H*-indole-3-carboxylate. Forensic Sci. Int..

[8] Cappadone C., Stefanelli C., Malucelli E., Zini M., Onofrillo C., Locatelli A., Rambaldi M., Sargenti A., Merolle L., Farruggia G. (2015). p53-dependent and p53-independent anticancer activity of a new indole derivative in human osteosarcoma cells. Biochem. Biophys. Res. Commun..

[9] Kaushik N.K., Kaushik N., Attri P., Kumar N., Kim C.H., Verma A.K., Choi E.H. (2013). Biomedical Importance of Indoles. Molecules.

[10] Estevão M.S., Carvalho L.C., Ribeiro D., Couto D., Freitas M., Gomes A., Fernandes L.M.F.E., Marques M.M.B. (2010). Antioxidant activity of unexplored indole derivatives: Synthesis and screening. Eur. J. Med. Chem..

[11] Ayati A., Emami S., Asadipour A., Shafiee A., Foroumadi A. (2015). Recent applications of 1,3-thiazole core structure in the identification of new lead compounds and drug discovery. Eur. J. Med. Chem..

[12] Rouf A., Tanyeli C. (2015). Bioactive thiazole and benzothiazole derivatives. Eur. J. Med. Chem..

[13] Chimenti F., Bolasco A., Secci D., Chimenti A., Granese A., Carradori S., Yáñez M., Orallo F., Ortuso F., Alcaro S. (2010). Investigations on the 2-thiazolylhydrazyne scaffold: Synthesis and molecular modeling of selective human monoamine oxidase inhibitors. Bioorg. Med. Chem..

[14] Nosrat O., Mahmoodi N.O., Khalili B., Rezaeianzade O., Ghavidast A. (2016). One-pot multicomponent synthesis of indol-3-ylhydrazinyl thiazoles as antimicrobial agents. Res. Chem. Intermed..

[15] Carradori S., Rotili D., de Monte C., Lenoci A., D’Ascenzio M., Rodriguez V., Filetici P., Miceli M., Nebbioso A., Altucci L. (2014). Evaluation of a large library of (thiazol-2-yl)hydrazones and analogues as histone acetyltransferase inhibitors: Enzyme and cellular studies. Eur. J. Med. Chem..

[16] Moldovan C.M., Oniga O., Parvu A., Tiperciuc B., Verite P., Pirnau A., Crisan O., Bojita M., Pop P. (2011). Synthesis and anti-inflammatory evaluation of some new acyl-hydrazones bearing 2-aryl-thiazole. Eur. J. Med. Chem..

[17] Grozav A., Gaina L.I., Pileczki V., Crisan O., Silaghi-Dumitrescu L., Therrien B., Zaharia V., Berindan-Neagoe I. (2014). The Synthesis and Antiproliferative Activities of New Arylidene-Hydrazinyl-Thiazole Derivatives. Int. J. Mol. Sci..

[18] Ignat A., Lovasz T., Vasilescu M., Fischer-Fodor E., Tatomir C.B., Cristea C., Silaghi-Dumitrescu L., Zaharia V. (2012). Heterocycles 27. Microwave Assisted Synthesis and Antitumour Activity of Novel Phenothiazinyl-Thiazolyl-Hydrazine Derivatives. Arch. Pharm. Chem. Life Sci..

[19] Zaharia V., Ignat A., Palibroda N., Ngameni B., Kuete V., Fokunang C.N., Moungang M.L., Ngadjui B.T. (2010). Synthesis of some *p*-toluenesulfonyl-hydrazinothiazoles and hydrazino-bisthiazoles and their anticancer activity. Eur. J. Med. Chem..

[20] Spartan’06. http://www.wavefun.com/.

[21] Hehre W.J. (2003). A Guide to Molecular Mechanics and Quantum Chemical Calculations.

[22] Stephens P.J., Devlin F.J., Chabulowski C.F., Frisch M. (1994). Ab initio calculation of vibrational absorbtion and circular dichroism spectra using density functional force fields. J. Phys. Chem..

[23] Yi W., Dubois C., Yahiaoui S., Haudecoeur R., Belle C., Song H., Hardre R., Reglier M., Boumendjel A. (2011). Refinement of arylthiosemicarbazone pharmacophore in inhibition of mushroom tyrosinase. Eur. J. Med. Chem..

[24] Nastasă C., Tiperciuc B., Duma M., Benedec D., Oniga O. (2015). New hydrazones bearing thiazole scaffold: Synthesis, characterization, antimicrobial, and antioxidant investigation. Molecules.

[25] Stratil P., Klejdus B., Kuban V. (2006). Determination of total content of phenolic compounds and their antioxidant activity in vegetables-evaluation of spectrophotometric methods. J. Agric. Food Chem..

[26] Espinoza M., Olea-Azar C., Speisky H., Rodríguez J. (2009). Determination of reactions between free radicals and selected Chilean wines and transition metals by ESR and UV–vis technique. J. Spectrochim. Acta A Mol. Biomol. Spectrosc..

[27] Mocan A., Crisan G., Vlase L., Crisan O., Vodnar D.C., Raita O., Gheldiu A.-M., Toiu A., Oprean R., Tilea I. (2014). Comparative Studies on Polyphenolic Composition, Antioxidant and Antimicrobial Activities of *Schisandrachinensis* Leaves and Fruits. Molecules.

